# The presynaptic machinery at the synapse of *C. elegans*

**DOI:** 10.1007/s10158-018-0207-5

**Published:** 2018-03-12

**Authors:** Fernando Calahorro, Patricia G. Izquierdo

**Affiliations:** Biological Sciences, University of Southampton, Life Sciences Building 85, Southampton, SO17 1BJ UK

**Keywords:** *C. elegans*, Synapse, Synaptic proteins, Synaptogenesis, Synaptic vesicles

## Abstract

Synapses are specialized contact sites that mediate information flow between neurons and their targets. Important physical interactions across the synapse are mediated by synaptic adhesion molecules. These adhesions regulate formation of synapses during development and play a role during mature synaptic function. Importantly, genes regulating synaptogenesis and axon regeneration are conserved across the animal phyla. Genetic screens in the nematode *Caenorhabditis elegans* have identified a number of molecules required for synapse patterning and assembly. *C. elegans* is able to survive even with its neuronal function severely compromised. This is in comparison with *Drosophila* and mice where increased complexity makes them less tolerant to impaired function. Although this fact may reflect differences in the function of the homologous proteins in the synapses between these organisms, the most likely interpretation is that many of these components are equally important, but not absolutely essential, for synaptic transmission to support the relatively undemanding life style of laboratory maintained *C. elegans*. Here, we review research on the major group of synaptic proteins, involved in the presynaptic machinery in *C*. *elegans*, showing a strong conservation between higher organisms and highlight how *C. elegans* can be used as an informative tool for dissecting synaptic components, based on a simple nervous system organization.

## Background

Transmission of a signal within a neuron is carried by depolarization of the resting membrane potential typically involving a transient reversal of the resting membrane potential. The depolarization leads to the opening of voltage-gated Ca^2+^ channels in the presynaptic membrane. Then, the opening of these channels causes a rapid influx of Ca^2+^ into the presynaptic terminal, facilitating synaptic vesicles fusion with the presynaptic plasma membrane of the neuron, and causing neurotransmitters to be released into the synaptic cleft. Neurotransmitters in the synaptic cleft diffuse away from the release site and have ready access to binding sites on synaptic receptors localized on both the post- and presynaptic membrane. They bind to their cognate receptors and elicit a transduction cascade to bring about the cellular response. In nerve terminals, neurotransmitters are packaged into synaptic vesicles (SVs) and released by Ca^2+^-induced exocytosis (Katz 1971, 1979; Sudhof 2004). A precise neuronal reaction requires that SVs are clustered adjacent to the release site or presynaptic active zone. Here the SVs are docked and held in contact with the cell membrane by the docking complex, where they are primed for fusion. Then, a depolarization induces the opening of Ca^2+^ channels, and the rising Ca^2+^ concentration stimulates SV-plasma membrane fusion. For cells to respond rapidly and reliably to incoming depolarizing potentials, they must maintain a sufficient supply of vesicles containing neurotransmitter close to the active zone where the content is released from the presynaptic neuron. However, there are neurons that use only graded voltage signals. These ‘non-spiking’ neurons that encode information as graded potentials typically have higher information rates compared to ‘spiking neurons’ (DiCaprio et al. 2007). Graded potentials are a consequence of the passive electrical property of the membrane and depolarizing potentials the result of a coordinated response (van Steveninck and Laughlin 1996). Finally, neurotransmitter release can occur by action potential-independent spontaneous vesicle fusion. Although for decades it was thought that spontaneous transmission was a consequence of ‘leaky’ synapse, recent data show that this alternative mechanism underpins signalling roles involve in synapse maturation and homoeostatic plasticity (Ramirez and Kavalali 2011).

Neurotransmitters are secreted from neurons by two types of vesicles that are classified by their size and appearance in electron micrographs. Small clear synaptic vesicles (SCV) (40–60 nm diameter) contain small molecule, so-called classical transmitters, such as glutamate, GABA and acetylcholine, that activate postsynaptic ionotropic receptors mediating fast synaptic transmission, and metabotropic receptors mediating a more slow and sustained transmission. Dense-core vesicles (DCVs) (60–120 nm diameter) are characterized by their electron dense appearance and larger diameter relative to SCVs, containing neuropeptides and biogenic amine neuromodulators such as serotonin and dopamine. DCVs dock at the plasma membrane but might be excluded from active zones (Hammarlund et al. 2008). There are similarities in the fusion machinery for both vesicle types, but also there are differences in the kinetics of exocytosis, docking localization and physiological regulation of release (Martin 2003; Rettig and Neher 2002), suggesting that there are proteins and mechanisms that are distinct for SCV- and DCV-mediated exocytosis.

**Fig. 1:**
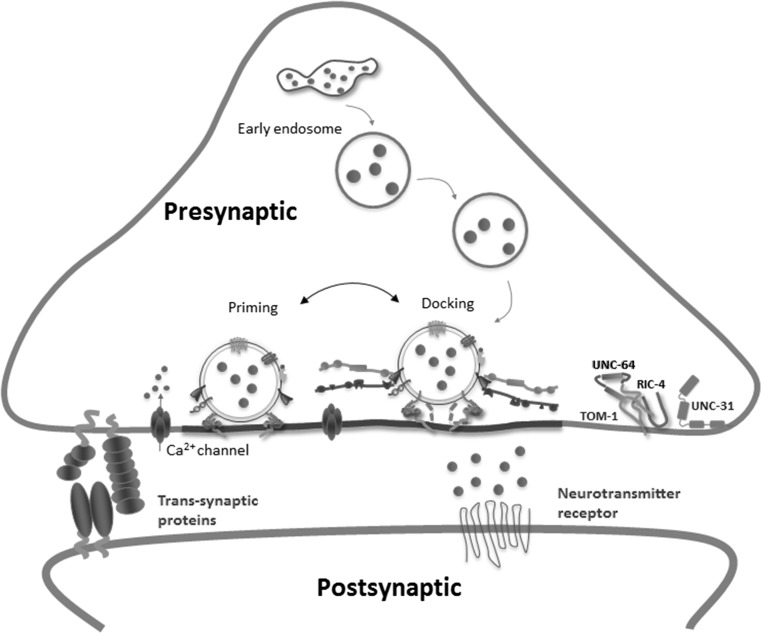
Molecular mechanisms of biogenesis and exocytosis of synaptic vesicles. Under resting conditions, synaptic vesicles are stored in the cytoplasm of the nerve terminal. Vesicles are loaded with neurotransmitter through an active processes requiring a neurotransmitter transporter and a vacuolar-type proton pump ATPase that provides a pH and electrochemical gradient. These transporters are selective for different classes of transmitters. The identity of many of these transporters was determined through the molecular characterization of *C. elegans* mutants. Filled vesicles dock at the active zone (represented by a thick grey line), where they undergo a priming reaction that makes them competent for Ca^2+^-triggered fusion-pore opening. Priming involves all steps required to acquire release preparation of the exocytosis complex. In special situations—i.e., during sustained activity, the priming could precede docking, resulting in immediate fusion of vesicles. After exocytosis, the vesicle proteins remain clustered in the plasma membrane to be recycled by endocytosis. The double arrow between docking and priming representations indicates that priming can precede docking instead to the interpretations based on ‘preferred’ models where docking is before priming. The last interpretation is supported by evidence, among others, such as *rab*-*3* and *unc*-*18* knockouts present an alteration in vesicle docking although the docking is not completely disrupted (Nonet et al. 1997; Weimer et al. 2003). Finally, synaptic vesicles are regenerated within the nerve terminal probably through one of the three proposed pathways (not shown in the diagram): a pathway in which vesicles endocytose by closure of the fusion pore and are refilled with neurotransmitters while remaining docked to the active zone (kiss-and-stay); a local recycling pathway that is clathrin independent but results in mixing vesicles with the reserve pool after endocytosis (kiss-and-run); and a pathway whereby vesicles undergo clathrin-mediated endocytosis and recycle either directly or via endosomes, ultrafast endocytosis removes membrane added by vesicle fusion at the lateral edge of the active zone. Large endocytic vesicles then fuse to endosomes, and in this way, newly formed synaptic vesicles can be recruited back to the active zone

Current models describing the molecular mechanism of Ca^2+^-regulated synaptic vesicle exocytosis and endocytosis divide the process into multiple steps, leading ‘preferred’ models: docking, priming, fusion, exocytosis (Fig. 1) (Jung and Haucke 2007; Sudhof 1995, 2004). This process is facilitated by the formation of a complex between molecules on the synaptic vesicle and molecules attached to the plasma membrane. Ca^2+^ binding to synaptotagmin triggers release by stimulating synaptotagmin binding to a molecular complex composed of SNARE (‘soluble NSF attachment receptor’) and SM (‘Sec1/Munc18-like’) proteins that mediates membrane fusion during exocytosis. Synaptic vesicles containing synaptotagmin are positioned at the active zone, the site of vesicle fusion, by a protein complex containing RIM proteins. RIM proteins simultaneously activate docking and priming of synaptic vesicles and recruit Ca^2+^ channels to active zones, thereby connecting within a single complex the primed synaptic vesicles to Ca^2+^ channels. This architecture allows direct flow of Ca^2+^ ions from Ca^2+^ channels to synaptotagmin, mediating tight millisecond coupling of a depolarization to neurotransmitter release. Influx of Ca^2+^ then leads to the rapid completion of membrane fusion and the release of the neurotransmitter (Rizo and Sudhof 2002). Finally, after fusion the vesicular components are recycled through endocytosis to replenish the synaptic vesicle pools. Three functional and morphological classes of vesicle pools have been assigned: the readily releasable pool (docked at active zones and ‘ready to go’ upon stimulation), the recycling pool (scattered throughout the nerve terminals and recycling upon moderate stimulation) and finally the reserve pool (occupying most of the vesicle clusters and only recycling upon C strong stimulation) (Sudhof 2013).

Key insights into the molecular mechanisms of synaptic events have come from research using genetic model systems such as the nematode *Caenorhabditis elegans* (Richmond 2005). Of particular note are studies employing mutants with uncoordinated locomotion (*unc* genes) (Brenner 1974) to define key synaptic determinants, optogenetics coupled with high-pressure freezing to resolve the relationship between docking and fusion (Watanabe et al. 2013) and genetic manipulation of syntaxin to define priming events (McEwen and Kaplan 2008). Here we provide a review of regulated exocytosis in *C. elegans* by presynaptic elements, highlighting *C. elegans* synapse as a powerful tool to dissect synaptic components and understanding key synaptic processes. We comment on the opportunity for future research directions deploying this model organism.

## Attachment of vesicles to the cytoskeleton

**Fig. 2:**
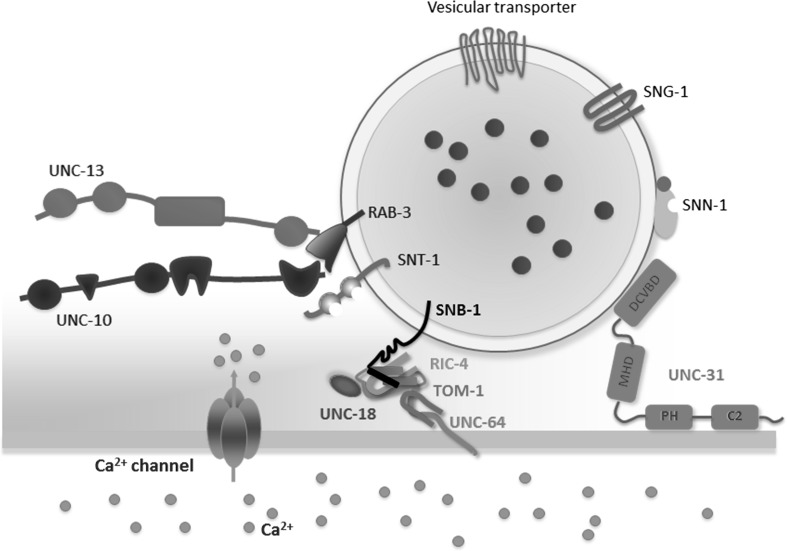
Molecular protein complexes that organize the secretory machinery at the presynaptic active zone. The vesicle clusters dock at the active zone through Rab proteins, CAPs protein (UNC-31), Munc-18 (UNC-18) and tomosyn. RIM (UNC-10) protein places the priming factor Munc-13 and Ca^2+^ channels into close proximity to synaptic vesicles and SNARE protein complex-dependent (synaptobrevin, SNAP-25, syntaxin) fusion machinery. In addition to Ca^2+^ channels, RIM proteins directly bind to the vesicle protein Rab3, to the priming factor Munc-13. Munc-13 directly activates the SNARE protein assembly. Both RIM and Munc-13 proteins are tightly regulated in a manner that determines presynaptic plasticity. The diagram is based on the Sudhof’s synaptic model (Sudhof 2013) and represents a magnified view of vesicle docking shown in Fig. 1

The principal synaptic protein which functions as a cytoskeleton anchor for vesicles in the reserve vesicle pool is synapsin (Fig. 2). Synapsins comprise a family of synaptic vesicle proteins that have been identified in a variety of invertebrate and vertebrate species (Stavoe et al. 2012; Cesca et al. 2010). In *C. elegans*, there is a homologue of synapsin protein (SNN-1) which is most similar to vertebrate synapsin II. In vertebrates, synapsins present highly conserved domains among the different isoforms. The best characterized domains are: domain A containing a phosphorylation site for PKA/CaMKI that regulates binding to synaptic vesicles, domain C containing ATP binding sites and domain E that regulates the reserve pool of synaptic vesicles. In *C*. *elegans, snn*-*1* presents a conserved domain organization with PKA/CaMKI site within domain A, several ATP binding sites in domain C and a highly conserved domain E (Cesca et al. 2010). *ssn*-*1* is expressed in neurons exhibiting patterns consistent with localization to vesicles in presynaptic regions. Very little is known about the exact role of SNN-1 in *C. elegans*, since *snn*-*1* mutants display predominantly wild-type phenotypes. However, a detailed analysis of specific synapses in *snn*-*1* mutants reveals synaptic vesicle clustering defects in the sensory neuron AIY (Stavoe et al. 2012) and resistance to paralysis on aldicarb (Sieburth et al. 2005). This latter phenotype is indicative of reduced acetylcholine release at the body wall neuromuscular junction as the paralysis is induced by accumulation of acetylcholine in the presence of the cholinesterase inhibitor aldicarb. This assay has been extensively deployed to resolve genetic determinants of cholinergic transmission in *C. elegans* (Mahoney et al. 2006).

The synaptic vesicle clustering in mammals is regulated by F-actin protein through two different pathways, one dependent on synapsins and the other independent (Nelson et al. 2013). In mammals, the synapsin family consists of at least 9 isoforms encoded by 3 distinct genes which are characterized by a mosaic of conserved and variable domains (Fornasiero et al. 2010). Specifically, the N-terminal portion of all synapsins is highly conserved, whereas the C-terminal portion is variable because of heterogeneous combinations of two different domains (Kao et al. 1999; Porton et al. 2011). Synapsins are vesicle proteins that link synaptic vesicles to the presynaptic cytoskeletal matrix by interacting with actin (Bahler and Greengard 1987; De Camilli et al. 1983; Li et al. 1995). They are expressed in neurons exhibiting patterns consistent with localization to vesicles in presynaptic regions (Sieburth et al. 2005), having multiple functions within presynaptic terminals, including anchoring of synaptic vesicles to the actin cytoskeleton, recruitment of them to a reserve pool and regulation of the fusion of SVs (De Camilli et al. 1990; Pieribone et al. 1995; Hilfiker et al. 1998). They are also implicated in neuronal development, synaptogenesis and maintenance of mature synapses (Ferreira et al. 2000).

Studies in mice show that mutants lacking synapsin I appear to develop normally and do not have gross anatomical abnormalities. However, in these mutants, the giant fibre terminals, in the CA3 area of the hippocampus, are significantly smaller, the number of synaptic vesicles is reduced, and the presynaptic structures altered (Takei et al. 1995). Furthermore, suppression of synapsin II leads to an inhibition of developing and synapse formation in hippocampal neurons. Similarly, a depletion of synapsin III affects the extension of processes and axon differentiation in hippocampal neurons (Bloom et al. 2003; Ferreira et al. 1995, 2000; Takei et al. 1995).

## Docking

The vesicle cluster that represents the reserve pool dock at the active zone through a subset of synaptic proteins include Rab proteins, CAPs protein (UNC-31), Munc-18 (UNC-18) and tomosyn (Fig. 2). Rab proteins present a key role regulating the recruitment of vesicles to the active zone in *C*. *elegans*. The Rab proteins are a large family of monomeric GTPases, conserved from yeast to humans, which through specific scaffolding with distinct interactors specify presynaptic function (Bock et al. 2001; Stenmark and Olkkonen 2001). In *C. elegans*, there are 31 members of the Rab family, 29 of which are also found in humans as orthologues (Gallegos et al. 2012). Probably, the most extensively studied Rab proteins in *C*. *elegans* are RAB-27/AEX-6 and RAB-3, homologues of human RAB-27 and RAB-3, respectively. *C*. *elegans rab*-*27* is expressed in neurons and in the intestine. In the nervous system, *rab*-*27* localizes to synapse-rich regions of the nervous system (nerve ring, dorsal and ventral cord) and partially co-localizes with synaptic vesicle-associated *rab*-*3*, although RAB-27 immunostaining is normal in *rab*-*3* mutants, suggesting that RAB-27 localization is independent of RAB-3 function (Mahoney et al. 2006). This expression profile is consistent with that of mammalian Rab27B, which is also expressed in both brain and intestine as well as other secretory cells. Both RAB-27 and RAB-3 present a key role in synaptic transmission process in *C*. *elegans*, regulating the recruitment of vesicles to the active zone or sequestration of vesicles near release sites (Mahoney et al. 2006). In vertebrates, Rab molecules regulate vesicular trafficking in many different transport pathways for both exocytosis and endocytosis in neural and non-neuronal tissues, but in *C. elegans* RAB-3 in neurons specifically plays a crucial role in regulating synaptic vesicle-mediated release (Nonet et al. 1997). As a consequence, *C. elegans rab*-*3* mutants present slight behavioural abnormalities. They are resistant to the paralytic action of the cholinesterase inhibitor aldicarb suggesting that cholinergic transmission is generally depressed (Mahoney et al. 2006; Nonet et al. 1997), as well as exhibiting an altered morphology of neuromuscular junctions (Nonet et al. 1997). There is a depletion of ≈ 40% of normal levels in vesicle population at synapses (identified by electron microscopy) accompanied by an elevation of these populations in inter-synaptic regions of the axons consistent with a deficit in SCV trafficking (Mahoney et al. 2006; Nonet et al. 1997). In addition, extracellular electrophysiological recordings reveal an impairment of synaptic transmission in the pharyngeal nervous system (Nonet et al. 1997). There is only one isoform of *rab*-*27* in *C. elegan*s, while two isoforms are found in mammals. Like the *rab*-*3* mutants, *C*. *elegans rab*-*27* mutants are slightly aldicarb resistant indicative of a reduction in cholinergic signalling, and exhibiting defecation defects consistent with neuromuscular transmission dysfunction in anterior body wall muscle contraction and expulsion steps of the defecation motor program (Mahoney et al. 2006).

In humans, the number of Rab genes reaches up to 60 where 33 of them have been identified by proteomic analyses in synaptic vesicle fractions (Takamori et al. 2006). Of these, Rab-3A, 3B, 3C, 3D and 27-B are involved in exocytosis, while Rab-4, 5, 10, 11B and 14 are intermediates of synaptic vesicle recycling such as early endosomes (Binotti et al. 2016). Specifically, Rab-3A and Rab-27B are the best investigated and play overlapping roles during Ca^2+^-triggered neurotransmitter release in mammals (Schluter et al. 2002).

Another indispensable actor for synaptic vesicle-mediated exocytosis is UNC-31. This is also known as CAPS (Ca^2+^-dependent activator protein for secretion) and is a multi-domain protein containing, from the N to the C terminus, a dynactin 1 binding domain (DBD), a C2 domain, a PH domain, a (M)UNC-13 homology domain (MHD) and finally a DCV binding domain (DCVBD) (Ann et al. 1997). The DBD is required for CAPS sorting (Sadakata et al. 2007b). The C2 domain, as a Ca^2+^ sensor, mediates Ca^2+^-dependent binding to phospholipids (Rizo and Sudhof 1998). The PH domain interacts with acidic phospholipids and binds with plasma membranes (Lemmon 2008). The MHD domain directly interacts with syntaxin, transforming syntaxin from the closed conformation into the open form giving the opportunity for syntaxin to form the SNARE core complex required for priming and docking (Basu et al. 2005; Betz et al. 1997; Hammarlund et al. 2008). Finally, the DCVBD domain mediates CAPS targeting to DCV (Grishanin et al. 2002).

CAPS was first identified as an essential protein for noradrenaline release from PC12 cells and recognized as being orthologous to *C. elegans* UNC-31 which had previously been shown to be involved in neurosecretion (Walent et al. 1992). As the name of the protein suggests, mutations in *unc*-*31* result in uncoordinated motor behaviour and the worms are constitutively lethargic with slow and soft movements (Avery et al. 1993). In addition, *unc*-*31* mutants feed constitutively and they have defects in egg laying and failures in recovery from dauer, a metabolically quiescent developmental larval stage of *C. elegans* (Avery et al. 1993; Dalliere et al. 2016). Consistent with this, the expression pattern of *unc*-*31* reveals a broad distribution in the nervous system (Charlie et al. 2006).

UNC-31/CAPS has been associated with exocytosis mediated by DCVs (Berwin et al. 1998), and in line with this, in *C. elegans* it has been found that loss of the single isoform of UNC-31 decreases neuropeptide secretion accompanied by an increase in neuropeptide abundance in motor axons (Sieburth et al. 2007). Mammals express two isoforms of *CAPS*, *CAPS1* and *CAPS2*, with similar functions but which differ in their spatiotemporal expression pattern (Sadakata et al. 2007c; Speidel et al. 2003). CAPS1 is essential for the uptake or storage of catecholamines in DCVs (Speidel et al. 2005), while CAPS2 appears to be required for DCV-mediated neurotrophin secretion in the cerebellum (Sadakata et al. 2007a, c). In addition, in *CAPS*-*1*/*CAPS*-*2* double null mutants DCV secretion is severely reduced (Farina et al. 2015). While early studies seemed to indicate that CAPS is not required for exocytosis of glutamate-containing SCVs (Tandon et al. 1998), this has been revised with further investigation that provides evidence for a more overlapping functional role with SCV-mediated exocytosis. *CAPS1* and *CAPS2* double knockout mice exhibit specific priming defects in glutamatergic transmission (Jockusch et al. 2007). In *Drosophila melanogaster*, in which a single gene encodes *dCAPS*, there is a ≈ 50% loss in evoked glutamatergic transmission at the neuromuscular junction, as well as an accumulation of synaptic vesicles at active zones (Renden et al. 2001).

Munc-18 proteins are the mammalian homologue of UNC-18 proteins in *C. elegans* and are a member of the Sec1/Munc18-like (SM) protein family. Munc-18 is a key synaptic protein acting during multiple stages of the exocytosis including vesicle priming, docking and fusion. Although during these steps syntaxin interactions are required, Munc-18 also regulates vesicle fusion via syntaxin-independent interactions. These syntaxin interactions are possible through a Munc-18 closed conformation of syntaxin binding. In *C. elegans*, UNC-18 is a protein required in neurons for synaptic vesicle-mediated exocytosis. Characterization of *unc*-*18* reveals a localization in ventral-cord motor neurons and some unidentified head neurons in the adult hermaphrodite (Gengyo-Ando et al. 1993). A similar expression is observed in males, but also a strong expression in the gonad (Schindelman et al. 2006). *unc*-*18* mutants are deficient in synaptic transmission with a reduction in neurotransmitter release and a consequent resistance to aldicarb (Graham et al. 2011; Gracheva et al. 2010). The introduction of a gain of function mutation in a functionally important domain (3b) within the UNC-18 protein confers a hypersensitivity to aldicarb (Graham et al. 2011). Munc-18 has a function in several exocytosis processes requiring syntaxin-dependent interactions; however, data based on *C*. *elegans* studies reveal a key role of domain 3b of Munc-18 in transducing regulation of vesicles fusion independent of closed-conformation syntaxin binding (Graham et al. 2011). This fact highlights *C*. *elegans* as a powerful and key tool to discover functional analysis of synaptic proteins, enhanced by the availability of CRISPR editing. Physiological data and electron micrographs of *C*. *elegans* neuromuscular junction provide evidence that in the absence of UNC-18 the size of the ready releasable pool of vesicles is drastically reduced (Weimer et al. 2003). Thus, *unc*-*18* mutants present a reduction in docked vesicles at the active zone, indicating that UNC-18 functions as a facilitator of vesicle docking (Weimer et al. 2003). Overall, the release defects in *unc*-*18* mutants are associated with the lack of two morphologically distinct vesicle pools: those tethered within 25 nm of the plasma membrane and those docked with the plasma membrane (Gracheva et al. 2010).

TOM-1 has also a role regulating the macromolecular complex binding between the SNARE proteins syntaxin, SNAP-25 and synaptobrevin, three synaptic molecules participating in the priming step. Tomosyn is a soluble protein first isolated from rat brain as a syntaxin binding partner capable of disrupting Munc18–syntaxin-1a complexes (Fujita et al. 1998). Tomosyn has two recognizable domains, an N-terminal domain rich in WD40 repeats and a C-terminal SNARE domain with high sequence homology to the R-SNARE domain of synaptobrevin (Hatsuzawa et al. 2003; Masuda et al. 1998). *C. elegans* tomosyn (TOM-1) is a cytosolic syntaxin binding protein implicated in the modulation of both constitutive and regulated exocytosis that negatively regulates synaptic vesicle priming in *C*. *elegans* (Gracheva et al. 2006). Thus, tomosyn inhibits synaptic vesicle priming through its synaptobrevin SNARE motif, which forms an inhibitory SNARE complex with syntaxin and SNAP-25 (McEwen et al. 2006). The expression of *tom*-*1* is observed in ventral nerve cord motor neurons and in a subset of neurons in head and tail ganglia (Dybbs et al. 2005). The synapses in *C. elegans tom*-*1* mutants present no changes in neuronal outgrowth or in synaptogenesis but exhibit prolonged evoked postsynaptic responses. This latter phenotype is accompanied by an increase in the number of plasma membrane-contacting vesicles (Gracheva et al. 2006). Thus, tomosyn-deficient mutants have increased synaptic transmission, an increased pool of primed vesicles and increased abundance of UNC-13 (a synaptic protein involved in Ca^2+^-triggered fusion-pore opening described in the next sections) at synapses (McEwen et al. 2006). This indicates that priming is negatively regulated by TOM-1 and that there is a fine balance between tomosyn and UNC-13, with the availability of open syntaxin a possible mechanism for this regulation (McEwen et al. 2006).

Overall, these findings from *C. elegans* along with studies using mouse models have shown tomosyn has a diffuse distribution in neurites and is accumulated at synapses co-localized with both moving SCVs and DCVs, regulating their mediated secretion. This suggests a function controlling the delivery, synaptic sharing and secretion of neuronal signalling molecules (Geerts et al. 2017).

## Priming

After the docking of vesicles at the active zone, they undergo a priming reaction regulated by a few key synaptic elements, syntaxins (UNC-64), synaptobrevin (SNB-1) and SNAP-25 (RIC-4) (Fig. 2). Nevertheless, there might be a regulatory-coupled process between docking and priming of the synaptic vesicles (Fig. 1); namely, synapses lacking priming proteins, such as Munc-13 or SNARE, have a reduced or absence docking of vesicles (Imig et al. 2014). Syntaxins are a family of transmembrane proteins that participate in SNARE complexes to mediate membrane fusion events including exocytosis in different compartments of the nervous system such as axons, the soma/dendrites or astrocytes. In *C. elegans*, the *unc*-*64* gene encodes syntaxin, a plasma membrane receptor for intracellular vesicles that is orthologous to vertebrate syntaxin 1A and *Drosophila* Syx1A. It is expressed in neural cells, especially in motor neurons and neurons constituting head ganglions (Ogawa et al. 1998; Yamashita et al. 2009). UNC-64 is required for normal locomotion and possibly also for insulin secretion and is an essential component of the core synaptic vesicle fusion machinery (McEwen and Kaplan 2008). UNC-64 interacts with UNC-13, UNC-18 and SNB-1/synaptobrevin (Sassa et al. 1999). Thus, it has been shown that loss of the N-terminal binding interaction between the syntaxin UNC-64 and the protein UNC-18 severely impairs neuromuscular synaptic transmission in *C. elegans*, resulting in an uncoordinated phenotype (Munson and Bryant 2009). In addition, *unc*-*64*–null mutants are unable to move and develop beyond the first larval stage (Saifee et al. 1998). *C. elegans unc*-*64* and mammalian syntaxin-1A are functional orthologues as shown by the observation that *unc*-*64*–null mutant worms expressing the mammalian syntaxin-1A wild type are able to move, grow and reproduce (Park et al. 2016). Another example of the power of *C*. *elegans* to further analyse, in this case, the structure/function relationship of syntaxin-1, is the recent discovery that syntaxin-1 N-peptide is critical when syntaxin adopts an ‘open’ conformation to bend towards Munc-18 (Park et al. 2016).

Similar to *C*. *elegans*, mammalian syntaxins present a typical domain organization where the N-terminal region contains two different motifs: a short N‐terminal peptide (‘N‐peptide’) that binds to Munc18‐1 (Dulubova et al. 2007), and a larger H_abc_‐domain that consists of an autonomously folded three‐helical bundle (Bracher and Weissenhorn 2004). Perhaps, the best-studied membrane–fusion complex is that mediating synaptic vesicle fusion through syntaxin 1A/1B (Teng et al. 2001). It has been suggested that STX1A and STX1B are functionally redundant. Thus, STX1A KO mice show a normal lifespan, and hippocampal neurons with normal neurotransmission, indicating that STX1B functionally compensates the function of STX1A (Fujiwara et al. 2006; Gerber et al. 2008). However, complete loss or partial loss of STX1B in mice caused a pre-weaning death, suggesting that STX1A and STX1B have differential functions (Mishima et al. 2014).

Synaptobrevins are vesicle-associated proteins involved in neurotransmitter release (Nonet et al. 1998). In *C. elegans*, SNB-1 is broadly present in nervous system, in neurons in the head ganglia and motor neurons in ventral nerve cord. Particularly, the abundance of SNB-1 in GABAergic motor neurons is controlled by MEC-15, one of a small number of F-box proteins evolutionarily conserved from *C*. *elegans* to mammal (Sun et al. 2013). *C. elegans* null *snb*-*1* mutants are not viable and die soon after hatching (Nonet et al. 1998). In an attempt to generate viable *C*. *elegans snb*-*1*-deficient mutants, the I97D substitution in *snb*-*1(e1563)* changes a hydrophobic residue to a charged residue in the TMD of synaptobrevin, leading to a synaptobrevin with reduction in function. *snb*-*1* mutants carrying this substitution are viable, with grossly normal locomotion (Sandoval et al. 2006). These mutants are resistant to the acetylcholinesterase inhibitor aldicarb, indicating that cholinergic transmission is impaired, and present abnormal electropharyngeograms which are extracellular recordings of the pharyngeal neuromuscular network (Nonet et al. 1998).

Studies on synaptobrevins in mouse are difficult to carry out since synaptobrevin 1 and 2 mutants, the two isoforms extensively expressed in the central nervous system (Schoch et al. 2001), immediately die after birth (Nystuen et al. 2007; Schoch et al. 2001). In this sense, *C*. *elegans* has been a useful tool dissecting the role of synaptobrevins in neurotransmitter release. However, using high-density cultures of hippocampal neurons from embryos, a drastic reduction in Ca^2+^-triggered vesicle fusion has been observed (Schoch et al. 2001).

The SNAP protein family consists of several homologous proteins of which SNAP-25 is essential for SV fusion (Delgado-Martinez et al. 2007). The *C. elegans* orthologue of vertebrate *SNAP*-*25*, *ric*-*4*, appears to be expressed selectively in the nervous system including the nerve ring, commissures, and ventral and dorsal nerve cords (Hwang and Lee 2003). Little is known about the role of *ric*-*4* at the *C*. *elegans* synapse; however, it is known that the loss of *ric*-*4* function via RNAi experiments leads to aldicarb resistance, indicating that *ric*-*4* plays a role in synapse structure and function (Sieburth et al. 2005). Studies using mouse models show that the deletion of SNAP-25 leads to reduced neuronal survival and impaired arborisation, reduced spontaneous release, and arrest of evoked release in the surviving neurons (Delgado-Martinez et al. 2007). In addition, the neurons of SNAP-25 null mutant mice (SNAP-25 KO) contain fewer DCVs and have reduced DCV fusion probability in surviving neurons at DIV14 (*days* in vitro). Others SNAP family members such us SNAP-23, SNAP-29 and SNAP-47 are also present in neurons and in synaptic vesicle purifications (Holt et al. 2006). Overexpression of SNAP-29 inhibits synaptic vesicle fusion possibly via inhibiting SNARE complex disassembly (Pan et al. 2005). Finally, SNAP-47 binds to plasma membrane SNAREs in vitro, but is predominantly located on intracellular membranes (Holt et al. 2006).

## Ion channel regulation and fusion

The priming reaction makes the vesicles competent for Ca^2+^-triggered fusion-pore opening. The major elements among the synaptic proteins involved in priming of SVs are synaptotagmin (SNT-1), synaptogyrin (SNG-1), Munc-13 (UNC-13) and RIM (UNC-10) (Fig. 2).

One key factor in Ca^2+^ regulation and vesicle fusion is the Ca^2+^ sensor synaptotagmin, which consists of a short N-terminal luminal segment, a single transmembrane α-helix, an unstructured linker, and two Ca^2+^ binding C2 domains, termed C2A and C2B, respectively. In *C*. *elegans*, the two synaptotagmin isoforms, *snt*-*1a* and *snt*-*1b*, are expressed in neurons, where *snt*-*1a* is typically expressed at higher levels and in a larger subset of neurons. In addition, *snt*-*1b* is exclusively expressed in the excretory duct cell and a subset of tail neurons including DVB, a GABAergic neuron required for defecation (Nonet et al. 1993; Mathews et al. 2007). Behaviourally, *snt*-*1* mutants present locomotory defects in swimming behaviour, as well as in the defecation motor program (Mathews et al. 2007). Evoked synaptic transmission is dependent on interactions between synaptotagmin and the SNARE complex, comprised of syntaxin, SNAP-25 and synaptobrevin. It has been shown that *snt*-*1* *C. elegans* mutants present a significant reduction in this evoked transmission, indicating its key role in SNARE complex assembly (Yu et al. 2013). On the other hand, *snt*-*1* mutants present large irregular cisternae associated with abnormal endocytosis, indicating a defect impacting at this level of the vesicle cycle (Yu et al. 2013). In addition, morphometric analyses of NMJ (neuromuscular junction) in *snt*-*1* mutants reveal a reduction in vesicle density, a phenotype associated with an endocytosis defect (Yu et al. 2013). *snt*-*1* mutants also show a reduction in absolute numbers of docked vesicle, and this docking defect appeared to be a consequence of an overall reduced vesicle density, since the fraction of docked vesicles as a function of total vesicles in *snt*-*1* mutants was not significantly reduced compared to wild type (Yu et al. 2013). Both reductions in absolute docked vesicles and in evoked transmission suggest that SNT-1 has additional function beyond exocytosis, consistent with the well-documented role of *snt*-*1* as a Ca^2+^ sensor promoting vesicle fusion (Yu et al. 2013). In addition, SNT-1 in *C. elegans* is crucial for the SVs (synaptic vesicles) association of RAB-3 protein. SNT-1 promotes the GTP-bound state of RAB-3 by inhibiting RAB-3 GAP, and thus, the catalytic subunit of RAB-3 GAP (RBG-1) localizes on SVs and directly binds to SNT-1 (Cheng et al. 2015). Ca^2+^ treatment disrupts the direct association between SNT-1 and RBG-1 (a Rab-3 GTPase). In addition, Ca^2+^ binding activity of SNT-1 is essential for the dissociation of RAB-3 from SVs (Cheng et al. 2015).

Complementary studies in mouse models have shown that synaptotagmins (specifically Syt1 and Syt2) are Ca^2+^ sensors for both synchronous and fast neurotransmitter release (Sun et al. 2007; Xu et al. 2009). Overall, synaptotagmins act as a cooperative Ca^2+^ receptor in exocytosis, binding Ca^2+^ at physiological concentrations. This binding is specific for Ca^2+^ and involves the cytoplasmic domain of synaptotagmin (Geppert et al. 1994; Pang et al. 2006).

Synaptogyrin and synaptophysin are tetraspan membrane proteins, the major vesicle proteins, characterized by four membrane-spanning domains that are tyrosine-phosphorylated (Arthur and Stowell 2007; Evans and Cousin 2005; Hubner et al. 2002). They are abundant and evolutionary conserved synaptic vesicle membrane proteins (Abraham et al. 2006) whose functions are poorly defined, and their depletion does not interfere with proper neuronal development and basic neuronal function in both *C. elegans* and mammals (Abraham et al. 2006; Eshkind and Leube 1995; McMahon et al. 1996). In contrast to vertebrates, in *C. elegans* the synaptogyrin but not the synaptophysin orthologue is predominant in neurons (Abraham et al. 2006; Hubner et al. 2002; Nonet 1999; Ruvinsky et al. 2007), expressed in all 26 GABAergic neurons, as well as in a subset of neurons across the nervous system (Abraham et al. 2011). In mouse and *C. elegans*, synaptogyrin is completely dispensable for nervous system development and performance of basic neuronal functions (Abraham et al. 2006; Eshkind and Leube 1995; McMahon et al. 1996). Thus, *C*. *elegans* mutants lacking or overexpressing synaptogyrin present an increased sensitivity to the epileptogenic GABA antagonist pentylenetetrazole (PTZ), showing a reduced convulsive threshold (Abraham et al. 2011). This suggests that modulation of the synaptic vesicle cycle is fine-tuned by the specific amount of synaptogyrin, since both decrease and increase in synaptogyrin result in an altered sensitivity to PTZ and aldicarb (Abraham et al. 2011). In addition, detailed analysis also uncovers mildly altered motility and decreased recruitment of synaptobrevin though not of RAB-3 to synapses, suggesting that synaptogyrin presents a distinct modulatory and redundant neuronal function in *C*. *elegans* (Abraham et al. 2011).

Another conserved core components of the presynaptic active zone are the UNC-13/Munc13 family. They are essential for both evoked and spontaneous SV release (Augustin et al. 1999; Richmond et al. 1999). These proteins contain multiple protein interaction domains and involved in many aspect aspects of presynaptic release. All the UNC-13/Munc13 isoforms contain a diacylglycerol binding C_1_ domain followed by a MUN domain including the MHD (Munc13 homology domain) flanked by C_2_B and C_2_C domains. The MUN domain, structurally similar to the vesicle tethering factors of the CATCHR (Complex Associated with Tethering Containing Helical rods) family (Li et al. 2011), is necessary for vesicle priming (Basu et al. 2005; Madison et al. 2005; Stevens et al. 2005) through binding to SNARE and Munc18 (Betz et al. 1997; Ma et al. 2011). The N-terminal regions of UNC-13/Munc13 isoforms are divergent in amino acid sequences and have been hypothesized to contribute to the distinct properties of SV exocytosis in different types of synapses (Augustin et al. 2001; Rosenmund et al. 2002).

In *C. elegans*, the *unc*-*13* locus produces two main isoforms that differ at the N-terminal region (Kohn et al. 2000). The expression of *unc*-*13* is in all neurons of both head and tail ganglia, as well as ventral nerve cord (Maruyama et al. 2001). Using genetic mutations that eliminate function of all isoforms or only UNC-13L demonstrate an essential role of UNC-13L in neurotransmitter release (Richmond et al. 1999). The kinetic components of release are thought to be mediated by SVs in different spatial domains of the nerve terminal. Rapid (or synchronous) release occurs within a few milliseconds and is proposed to consist of fusion of SVs that are close to Ca^2+^ entry sites. However, slow release occurs over tens to hundreds of milliseconds and is thought to be mediated by fusion of SVs that are farther from Ca^2+^ channels (Neher and Sakaba 2008). In *C. elegans*, the UNC-13L isoform is co-localized at presynaptic terminals concentrated near dense projections. By contrast, the UNC-13S isoform presents a diffuse distribution in axons. This suggests that UNC-13L and UNC-13S may mediate different forms of release and distinct effects on synaptic transmission (Hu et al. 2013). In this sense, it has been shown that UNC-13L is involved in both fast and slow release of SVs, while the short isoform UNC-13S is required for the slow release (Hu et al. 2013). In more detail, other studies using a unique *unc*-*13* mutant that specifically deletes the C_2_A domain of UNC-13L show that the precise position of UNC-13 in the active zone depended on the C_2_A domain. In addition, the C_2_A domain regulates the release probability of SVs, likely through positioning UNC-13L to the active zone, and that this domain also has a significant influence in spontaneous release (Zhou et al. 2013). Importantly, early works using *C*. *elegans* have shown the role of PKC/DAG in the modulation of UNC-13. Thus, the binding of DAG by UNC-13 drives its membrane association and regulates the exocytosis function of UNC-13 (Lackner et al. 1999). Later, studies in *Drosophila* have also shown how the PLC and DAG modulation as well as G-proteins regulates the synaptic levels of DUNC-13, critical determinant of SV fusion probability (Aravamudan and Broadie 2003).

RIM proteins are presynaptic scaffolding proteins specifically localized to the active zone and found to bind several presynaptic proteins, like Munc13-1, Rab3a and voltage-gated Ca^2+^ channels (Betz et al. 2001; Kaeser et al. 2011; Schoch et al. 2002; Wang et al. 1997). RIM proteins are encoded by four genes (*Rims1*–*4*); the *Rim1* and *2* genes give rise to five RIM isoforms, called RIM1α, RIM1β, RIM2α, RIM2β and RIM2γ (Kaeser et al. 2008; Wang and Sudhof 2003). Studies using RIM1α constitutive knockout mice and RIM mutants in *C. elegans* found roles for RIM1 in transmitter release, presumably via determining readily releasable vesicle pool size (Calakos et al. 2004; Koushika et al. 2001; Schoch et al. 2002). RIM mutants isolated in *C. elegans* (*unc*-*10*) are viable but exhibit behavioural and pharmacological defects, indicative of synaptic dysfunction according to the localization of RIM at the active zone. Although RIM was originally identified as a RAB-3 binding partner, the consequence of a loss of function mutation is more severe than *rab*-*3* mutants, suggesting that it possesses additional functions (Gracheva et al. 2008). Electrophysiological analysis of *unc*-*10* worms revealed both reduced evoked release of SVs and spontaneous synaptic event frequency, thus implicating RIM in release (Koushika et al. 2001). UNC-10 is co-localized with the Ca^2+^ channel, UNC-2 at *C. elegans* presynaptic densities and synaptic release in *unc*-*10* and *rab*-*3* mutants exhibit reduced Ca^2+^ sensitivity (Gracheva et al. 2008a).

## Concluding remarks

Through this review, we discussed the synaptic release machinery, and how the powerful genetic model *C*. *elegans* contributes to elucidating core processes of synaptic transmission. This is facilitated by the ability to maintain viable mutants of *C. elegans* for synaptic proteins that are otherwise essential in mice. It has broad relevance as *C. elegans* harbours the same elaborate elements for neurotransmission as mammals, and thus, it has been instrumental in key discoveries relating to the synaptic vesicle cycle. However, some key processes have yet to be elucidated; for example, the precise physicochemical mechanisms underlying fusion, the roles of key synaptic proteins with overlapping functions within complex neural networks and understanding how synaptic vesicles recaptured by clathrin-mediated endocytosis are placed back into the vesicle pool; all these aspects demand further study which may be supported by the *C. elegans* model. Advances in techniques to study the vesicle cycle in the intact living synapse in combination with genetic manipulation will accelerate progress in the field, shedding more light on these intricate processes. In this sense, *C*. *elegans* is an excellent system to facilitate discovery in this field, thanks to a simple, genetically tractable nervous system that is evolutionarily conserved with mammalian neurons, as well as providing new routes to understand the dynamic processes underlying neurotransmitter exocytosis.
